# Silicon decreases both uptake and root-to-shoot translocation of manganese in rice

**DOI:** 10.1093/jxb/erv545

**Published:** 2016-01-04

**Authors:** Jing Che, Naoki Yamaji, Ji Feng Shao, Jian Feng Ma, Ren Fang Shen

**Affiliations:** 1 State Key Laboratory of Soil and Sustainable Agriculture, Institute of Soil Science, Chinese Academy of Sciences, Nanjing, China; 2 University of Chinese Academy of Sciences, Beijing, China; 3 Institute of Plant Science and Resources, Okayama University, Chuo, Kurashiki, Japan

**Keywords:** ^54^Mn, Mn-Si complex, Mn toxicity, Mn transporter, root-to-shoot translocation, silicon

## Abstract

Silicon (Si) is known to alleviate manganese (Mn) toxicity in a number of plant species; however, the mechanisms responsible for this effect are poorly understood. Here, we investigated the interaction between Si and Mn in rice (*Oryza sativa*) by using a mutant defective in Si uptake. Silicon alleviated Mn toxicity in the wild-type (WT) rice, but not in the mutant exposed to high Mn. The Mn concentration in the shoots was decreased, but that in the roots was increased by Si in the WT. In contrast, the Mn concentration in the roots and shoots was unaffected by Si in the mutant. Furthermore, Si supply resulted in an increased Mn in the root cell sap, decreased Mn in the xylem sap in the WT, but these effects of Si were not observed in the mutant. A short-term labelling experiment with ^54^Mn showed that the uptake of Mn was similar between plants with and without Si and between WT and the mutant. However, Si decreased root-to-shoot translocation of Mn in the WT, but not in the mutant. The expression of a Mn transporter gene for uptake, *OsNramp5*, was unaffected by a short exposure (<1 d) to Si, but down-regulated by relatively long-term exposure to Si in WT. In contrast, the expression of *OsNramp5* was unaffected by Si in the mutant. These results indicated that Si-decreased Mn accumulation results from both Si-decreased root-to-shoot translocation of Mn, probably by the formation of Mn-Si complex in root cells, and uptake by down-regulating Mn transporter gene.

## Teaser

Silicon alleviated manganese toxicity in rice by down-regulating both manganese transporter expression for uptake and manganese root-to-shoot translocation.

## Introduction

Silicon (Si) is a beneficial element for plant growth and development (Ma and Yamaji, 2015). The beneficial effects of Si are characterized by enhancing the tolerance of plants to various abiotic and biotic stresses (Epstein, 1999; Ma, 2004; Ma and Yamaji, 2006). One of them is the alleviation of Mn toxicity, which is one of the major factors limiting plant growth in acid soils and insufficiently drained soils (Marschner, 1995; Pontigo *et al.*, 2015). The mechanisms for the Si-decreased Mn toxicity seem to differ with plant species. In rice, a typical Si-accumulating plant, Si alleviated Mn toxicity by decreasing Mn accumulation in the shoots (Okuda and Takahashi, 1962a; Horiguchi, 1988; Li *et al.*, 2012). A previous study showed that this might be attributed to the decreased Mn uptake by Si (Okuda and Takahashi, 1962a). Silicon promoted Mn oxidizing capacity of the roots in rice, resulting in oxidation of plant-available Mn^2+^ on the root surface, thereby suppressing uptake of Mn. Horiguchi (1988) reported that in addition to Si-decreased Mn uptake, Si also enhanced the internal tolerance to high Mn concentration. This is based on the finding that the concentration of Mn showing toxicity symptoms (brown spots on the leaves) was lower in the plants without Si than those with Si. Recently, Li *et al.* (2012) found that although Si enhanced Mn tolerance in both Mn-sensitive and -tolerant rice cultivars, the effect of Si on Mn accumulation differed between cultivars. In a Mn-sensitive rice cultivar, Si increased Mn in the roots, but did not affect Mn in the shoots. By contrast, Si decreased Mn of both the roots and shoots in a Mn-tolerant rice cultivar (Li *et al.*, 2012). Therefore, the exact mechanisms for the Si-increased Mn tolerance in rice remain unknown.

However in some other plant species – including barley (*Hordeum vulgare*), common bean (*Phaseolus vulgaris*), cowpea (*Vigna unguiculata*), cucumber (*Cucumis sativas*) and pumpkin (*Curcurbita pepo*) – the Si-increased Mn tolerance is not caused by reducing Mn accumulation but by alteration of the internal distribution of Mn. In barley and common bean, Si application resulted in a homogenous distribution of Mn in the leaves (Williams and Vlamis, 1957; Horst and Marschner, 1978; Horiguchi and Morita, 1987). By constrast, Si caused a localized accumulation of Mn together with Si in a metabolically inactive form at the base of trichomes of the leaf surface in pumpkin (Iwasaki and Matsumura, 1999). In cowpea and cucumber, it seems that Si affected the oxidative process of excess Mn mediated by peroxidase (POD) through the interaction with phenolic substances in the solution phase of the apoplast (Iwasaki *et al.*, 2002a, b; Dragisic Maksimovic *et al.*, 2007; Fuehrs *et al.*, 2009). In cucumber, Si increased the binding capacity of the cell wall to Mn, thereby lowering Mn concentration within the symplast (Rogalla and Romheld, 2002) and decreasing the free leaf apoplastic Mn^2+^ as a catalyst for the Fenton reaction (Dragisic Maksimovic *et al.*, 2012).

In the present study, we further investigated the interaction between Si and Mn in rice by using a mutant (*lsi1*) defective in Si uptake (Ma *et al.*, 2002). This mutant has a mutation in the influx transporter (Lsi1) of Si (Ma *et al.*, 2006). Comparison between the mutant and its wild-type rice revealed that Si-enhanced Mn tolerance results from both decreased Mn uptake, by down-regulating the Mn transporter gene, and reduced root-to-shoot translocation, probably due to the interaction of Si-Mn in root cells.

## Materials and methods

### Plant material and growth conditions

A rice (*Oryza sativa*) mutant (*lsi1*) and its wild type (cv. Oochikara, WT) were used in this study. The *lsi1* mutant was isolated and characterized previously (Ma *et al.*, 2002). Seeds were soaked in water overnight at 30°C in the dark and then transferred on to a net floating on a 0.5mM CaCl_2_ solution. After 7 d, the seedlings were transferred to a 3.5-l plastic pot containing half-strength Kimura B solution (pH 5.6) (Ma *et al.*, 2002). This solution was renewed every 2 d. The plants were grown in a greenhouse at 25–30°C under natural light. All experiments were conducted with three to four biological replicates.

### Effect of Si and Mn on the growth of rice

Seedlings (18-d-old) of both WT and *lsi1* mutant were grown in a nutrient solution containing 0.5 μM (low) and 200 μM (high) Mn without or with 1mM silicic acid. Silicic acid was prepared by passing potassium silicate through a cation-exchange resin (Amberlite IR-120B, H^+^ form) as described previously (Ma *et al.*, 2002). After 2 weeks, the roots and shoots were separately collected, the roots were washed with 5mM CaCl_2_ three times and separated from the shoots. The samples were dried at 70°C for 3 d and their dry weight was recorded. These samples were subjected to mineral determination as described below.

### Distribution of Mn in different leaves

Seedlings (18-d-old) of the rice *lsi1* mutant and its WT were cultured in a nutrient solution containing 200 μM Mn without or with 1mM silicic acid. After 10 d, an individual leaf from each of the leaf positions 2–8 was sampled for Mn determination.

### Mn concentration in root cell sap and xylem sap

Seedlings (28-d-old) of both the WT and *lsi1* mutant were exposed to a nutrient solution containing 5 μM Mn without or with 1mM Si as silicic acid. After 24h, the xylem sap and root cell sap were collected. For collection of xylem sap, the shoot (2cm above the root) was excised with a razor, and then the xylem sap was collected with a micropipette for 1h after decapitation of the shoot. For collection of root cell sap, the excised roots were placed on a filter in a tube and frozen at −80°C overnight. After thawing at room temperature, the cell sap was collected by centrifugation at 20 600 ×*g* for 10min. The residue (cell wall) was washed three times (5min for each washing) with 70% ethanol, then dried at 70°C for 3 d. A time-dependent change of Mn in the root cell sap was also investigated by exposing the WT and *lsi1* mutant (28-d-old) to a solution containing 5 μM Mn with/without 1mM Si as silicic acid. At 3, 6, 9, 12 and 24h after the exposure, the root cell sap was collected as described above.

### Mn and Si concentration determination

For determination of Mn concentration, the dried samples were digested with concentrated HNO_3_ (60%) at a temperature of up to 140°C. The Mn concentration in the digest solution, xylem sap and root cell sap, and the Si concentration in the root cell sap were determined by inductively coupled plasma-mass spectrometer (7700X; Aglient Technologies) after appropriate dilution.

### Short-term uptake with radioisotope-labelled Mn

Seedlings (30-d-old) of WT and *lsi1* mutant were pretreated with 5 μM Mn for 3 d. Four plants each were exposed to a 50-ml nutrient solution containing ^54^Mn (80 MBq µmol^-1^) with 5 μM cold Mn without/with 1mM Si. At 5, 10, 30, 60 and 180min after the exposure, the roots were washed with half-strength Kimura B solution (without ^54^Mn) three times and separated from the shoots. The sampled roots and shoots were subjected to radioactivity measurement. Radioactivity of each part was measured immediately using the auto-well gamma system ARC-360 (Aloka). To investigate the effect of Si pretreatment on Mn uptake and accumulation, WT seedlings (27-d-old) were first exposed to 1mM Si in the nutrient solution for 3 d and then subjected to the uptake experiment as described above.

### *Expression of the* OsNramp5 *gene*

To investigate the effect of Si on expression of *OsNramp5*, seedlings of the WT and *lsi1* mutant (18-d-old) were exposed to 0.5 and 200 μM Mn without/with 1mM Si for up to 3 d. The roots were sampled for RNA extraction at different times (1 and 3 d). Total RNA was extracted using an RNeasy Plant Mini Kit (Qiagen) and converted to cDNA using ReverTra Ace qPCR RT Master Mix with gDNA remover (TOYOBO) following the protocol supplied by the manufacturer. Specific cDNAs were amplified by SsoFast EvaGreen Supermix (Bio-Rad) and quantitative real-time PCR was performed on CFX384 (Bio-Rad). Primer sequences for Os*Nramp5* were 5′-CAGCAGCAGTAAGAGCAAGATG-3′ (forward) and 5′-GTGCTCAGGAAGTACATGTTGAT-3′ (reverse). *HistoneH3* was used as an internal standard with primer pairs 5′-AGTTTGGTCGCTCTCGATTTCG-3′ (forward) and 5′-TCAACAAGTTGACCACGTCACG-3′ (reverse). The relative expression was normalized by the ΔΔCt method using the CFX Manager software (Bio-Rad).

### Statistical analysis

Significance of differences between means was analyzed by Duncan’s test and Student’s *t*-test.

## Results

### Si alleviated Mn toxicity in wild-type rice, but not in the mutant

**Fig. 1.**
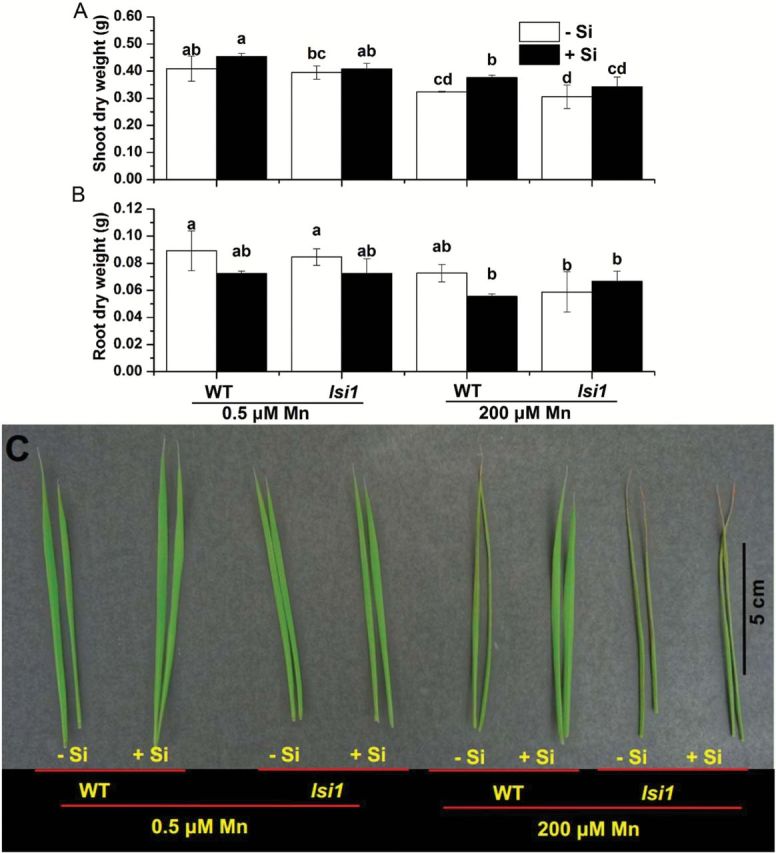
Effect of Si on Mn toxicity in rice. (A) Shoot and (B) root dry weight. (C) Mn toxicity symptoms of the old leaf (leaf 3). Seedlings (18-d-old) of mutant *lsi1* and its wild type (WT, cv. Oochikara) were cultured in a nutrient solution containing 0.5 (low) and 200 (high) μM Mn without or with 1mM silicic acid for 2 weeks. Data represent the mean ±SD (*n*=3). Different letters indicate a significant difference (*P*<0.05) using Duncan’s test.

We first compared the growth between the rice *lsi1* mutant and its WT at low (0.5 μM) and high (200 μM) concentration of Mn in the presence or absence of Si (1mM). At low Mn concentration, there was no difference in the growth of the roots and shoots between plants supplied with and without Si in both WT and *lsi1* mutant after 2 weeks’ growth (Fig. 1A). However, at high Mn, the shoot dry weight of both the WT and *lsi1* mutant was inhibited by 21.8% and 22.8%, respectively, in the absence of Si (Fig. 1A). The symptoms of Mn toxicity (brown spots) appeared in the old leaves (Fig. 1C). The effect of high Mn on root growth was hardly observed (Fig. 1B). However, Si addition improved the shoot growth in the WT, but not in the mutant (Fig. 1A). The toxicity symptoms disappeared in the WT leaves, but not in the mutant in the presence of Si at high Mn (Fig. 1C).

### Si decreased Mn accumulation in the shoots, but increased Mn accumulation in the roots of WT

**Fig. 2.**
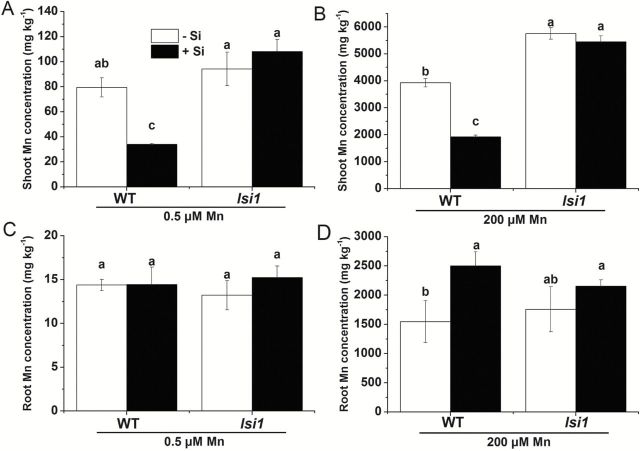
Effect of Si on Mn concentration in shoot and root of WT and *lsi1* mutant. Seedlings (18-d-old) of *lsi1* mutant and its wild type (WT, cv. Oochikara) were cultured in a nutrient solution containing 0.5 (low) and 200 (high) μM Mn without or with 1mM silicic acid for 2 weeks, and the shoots (A, B) and roots (C, D) were subjected to Mn determination. Data represent the mean ±SD (*n*=3). Different letters indicate a significant difference (*P*<0.05) using Duncan’s test.

At low Mn, shoot Mn concentration was decreased by Si supply in the WT, but not in the mutant (Fig. 2A). There was no effect of Si on the root Mn concentration in both WT and the mutant (Fig. 2C). By contrast, at high Mn, Si decreased Mn concentration in the shoots and increased Mn concentration in the roots in the WT (Fig. 2B, D). However, the Mn concentration of both the roots and shoots were unaffected by Si in the mutant (Fig. 2B, D).

**Fig. 3.**
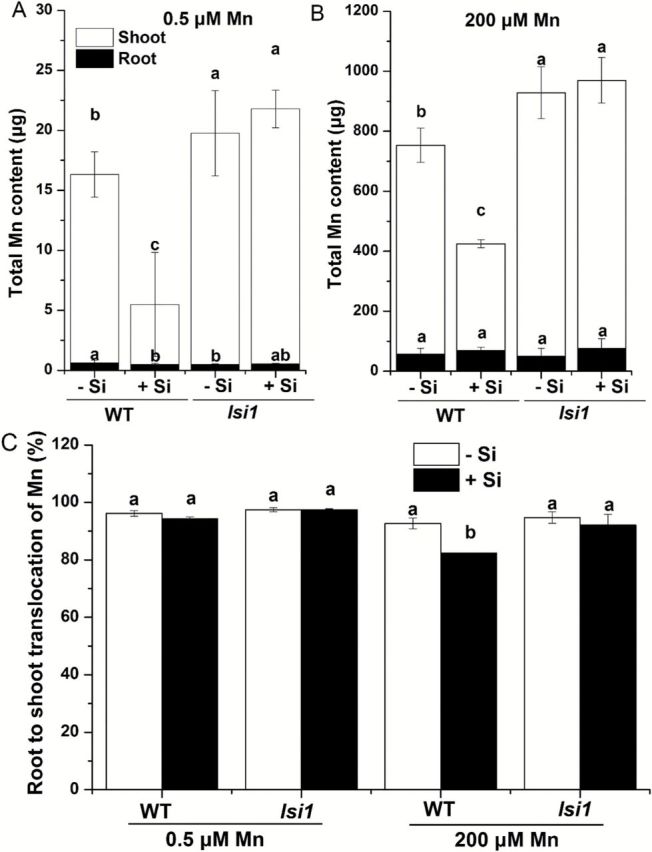
Effect of Si on uptake and translocation of Mn. (A, B) Mn content of WT and *lsi1* mutant at both low and high Mn supply. (C) Root-to-shoot translocation of Mn in WT and *lsi1* mutant at both low and high Mn supply. Seedlings (18-d-old) of *lsi1* mutant and its wild type (WT, cv. Oochikara) were cultured in a nutrient solution containing 0.5 (low) and 200 (high) μM Mn without or with 1mM silicic acid for 2 weeks. Data represent the mean ± SD (*n*=3). Different letters indicate a significant difference (*P*<0.05) using Duncan’s test.

The total content of Mn was decreased by Si in the WT by 66.3 and 43.6%, respectively, at low and high Mn (Fig. 3A, B). However, the Mn content was not affected by Si in the mutant at both low and high Mn supply. The root-to-shoot translocation of Mn was 92.6% in WT without Si, but decreased to 82.4% by Si at high Mn (Fig. 3C). Silicon did not affect the root-to-shoot translocation in the mutant, which was ~95% at both low and high Mn supply (Fig. 3C).

### Si did not alter Mn distribution in different leaves

**Fig. 4.**
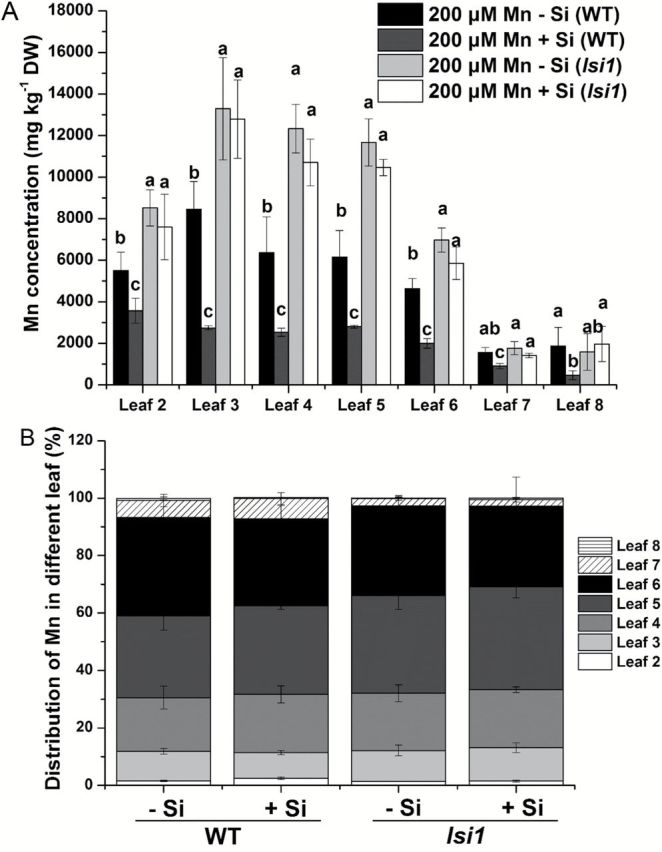
Effect of Si on Mn distribution in different leaves. Seedlings (18-d-old) of *lsi1* mutant and its wild type (WT, cv. Oochikara) were cultured in a nutrient solution containing 200 μM Mn without or with 1mM silicic acid for 10 d. (A) Mn concentration in different leaves; (B) distribution of Mn in different leaves. Percentage of Mn accumulation in each leaf/total Mn accumulation in the shoots is shown. Data represent the mean ±SD (*n*=4). Different letters indicate a significant difference (*P*<0.05) using Duncan’s test.

To examine the effect of Si on the distribution of Mn in different leaves, both the WT and mutant were grown in 200 µM Mn for 10 d in the presence and absence of Si. In all leaves, Si decreased Mn concentration in the WT, but not in the mutant (Fig. 4A). Mn concentration in the old leaves (leaves 2–6) was higher in the mutant than in the WT, but was similar in the new leaves (leaves 7–8) in the absence of Si (Fig. 4A). However, the presence of Si did not alter the Mn ratio between the leaves, either in WT or the mutant (Fig. 4B).

### Short-term uptake experiment with radioisotope ^54^Mn

**Fig. 5.**
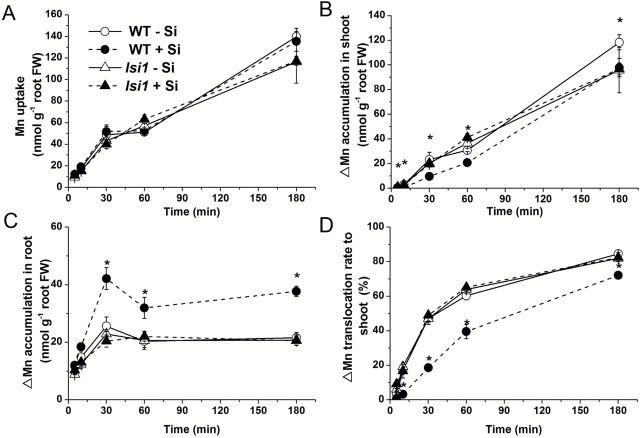
Short-term Mn uptake and translocation. Seedlings (30-d-old) of the WT and *lsi1* mutant were exposed to a nutrient solution containing 5 µM Mn labelled with ^54^Mn in the absence (-Si) or presence of 1mM (+Si) silicic acid for up to 180min. (A) Short-term Mn uptake; (B) ∆Mn accumulation in the shoots; (C) ∆Mn accumulation in the roots; (D) translocation rate of ∆Mn to the shoots. Data represent the mean ±SD (*n*=4). Asterisks indicate a significant difference (*P*<0.01) between WT -Si and WT +Si at a given time using Student’s *t*-test.

A short-term (up to 180min) uptake experiment of Mn was conducted using radioisotope ^54^Mn. In both the WT and the mutant, short-term Mn uptake increased with exposure time (Fig. 5A) and coexistence of Si with Mn in the solution did not affect the short-term uptake of Mn (Fig. 5A). However, coexistence of Si increased ∆Mn (net increase of Mn) accumulation in the roots, but decreased ∆Mn accumulation in the shoots of the WT (Fig. 5B, C), resulting in a decreased root-to-shoot translocation of ∆Mn (Fig. 5D). By contrast, neither the ∆Mn accumulation in the roots nor in the shoots was affected by Si in the mutant (Fig. 5).

**Fig. 6.**
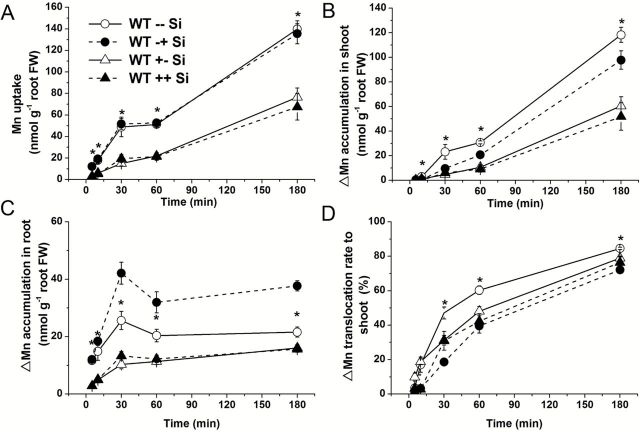
Effect of Si pretreatment on short-term Mn uptake and translocation. Seedlings (27-d-old) of the WT were grown in a nutrient solution with (+Si) or without (-Si) 1mM Si. After 3 d, the seedlings were exposed to a nutrient solution containing 5 µM Mn labelled with ^54^Mn and 0 (--, +-Si) or 1mM (-+, ++) of silicic acid for up to 180min. (A) Short-term Mn uptake; (B) ∆Mn accumulation in the shoots; (C) ∆Mn accumulation in the roots; (D) translocation rate of ∆Mn to the shoots. Data represent the mean ±SD (*n*=4). Asterisks indicate a significant difference (*P*<0.01) between WT --Si and WT +-Si at individual time using Student’s *t*-test. No significant difference (*P*<0.05) was found between WT ++Si and WT +-Si at any given time using Student’s *t*-test. For comparison, data without pretreatment from Fig. 5 are also shown.

Furthermore, to investigate the effect of pretreatment with Si on Mn accumulation, WT pretreated with/without Si for 3 d were subjected to the short-term ^54^Mn experiment as described above. Pretreatment with Si significantly decreased short-term Mn uptake, ∆Mn accumulation in the roots and shoots, and root-to-shoot translocation, irrespectively of coexistence of Si (Fig. 6). Coexistence of Si in the external solution during uptake period did not affect the uptake, accumulation in the roots and shoots and translocation of Mn (Fig. 6). This may be attributed to the Si remaining in the root cells during pretreatment.

### Si increased Mn in the root cell sap, but decreased Mn in the xylem sap

**Fig. 7.**
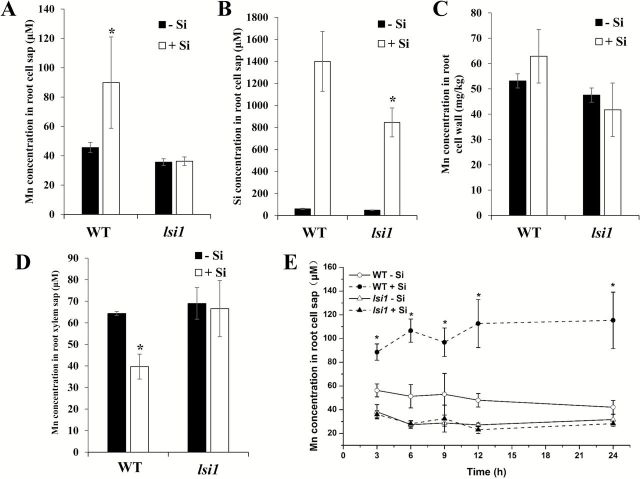
Effect of Si on Mn concentration in root cell sap and xylem sap. (A) Mn concentration in root cell sap; (B) Si concentration in root cell sap; (C) Mn concentration in the root cell wall; (D) Mn concentration in xylem sap. WT and *lsi1* seedlings (28-d-old) were exposed to a nutrient solution containing 5 μM Mn without or with 1mM Si for 24h. (E) Time-dependent change of Mn in the root cell sap. WT and *lsi1* seedlings (28-d-old) were exposed to a nutrient solution containing 5 μM Mn without or with or 1mM Si for up to 24h. Data represent the mean ±SD (*n*=3). Asterisks indicate significant difference (*P*<0.05) between -Si and +Si by Student’s *t*-test.

Mn concentration in the root cell sap and xylem sap was compared between plants supplied with and without Si in both WT and the *lsi1* mutant. After 24-h exposure to Si, the Mn concentration in the root cell sap was increased 2.0-fold by Si in the WT (Fig. 7A), but did not change in the mutant. The Si concentration in the root cell sap was much higher in WT than in the mutant (Fig. 7B). However, the Si:Mn stoichiometric ratio was about 25 in both WT and the *lsi1* mutant. In the root cell wall there was no difference in Mn accumulation between WT and the mutant (Fig. 7C) and here the presence of Si had no effect. The share of Mn in the root cell sap was ~32% in both WT and *lsi1* mutant in the absence of Si. However, in the presence of Si, the share of Mn in the root cell sap was increased to ~38% in WT, but was unchanged in the mutant. The Mn concentration in the xylem sap was decreased by Si in WT (Fig. 7D), but was not affected in the mutant. A time-course experiment further showed that Si-increased Mn in the root cell sap occurred as early as 3h after exposure to Si in the WT (Fig. 7E). Again the effect of Si on Mn in the root cell sap was not observed in the mutant (Fig. 7E).

### Si down-regulated the expression of Mn transporter, OsNramp5

**Fig. 8.**
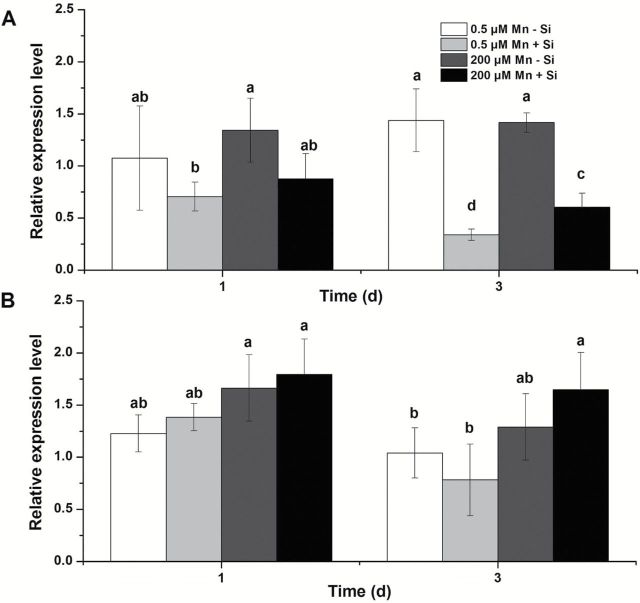
Effect of Si on expression of *OsNramp5* in the roots. Seedlings of WT (A) and *lsi1* mutant (B) were exposed to 0.5 or 200 μM Mn without or with 1mM silicic acid for up to 3 d. The expression of *OsNramp5* in the roots was determined by quantitative real-time PCR. *Histone H3* was used as the internal standard. Expression relative to WT (0.5 μM Mn -Si) is shown. Data are means ±SD of three biological replicates. Different letters indicate a significant difference (*P*<0.05) using Duncan’s test.

Mn uptake in rice is mediated by a transporter belonging to the Nramp family, OsNramp5 (Sasaki *et al.*, 2012). We compared the expression level of *OsNramp5* in plants exposed to Si or not, in both the WT and *lsi1* mutant. At day one after exposure, the expression level of *OsNramp5* was similar in plants with and without Si in the WT and *lsi1* mutant (Fig. 8). There was also no difference in the expression level between plants exposed to different Mn concentrations (0.5 μM vs. 200 μM). However, at day three, the expression level of *OsNramp5* was down-regulated by Si at both Mn concentrations in the WT, but not in the mutant (Fig. 8).

## Discussion

A number of studies have consistently shown that Si is able to alleviate Mn toxicity in rice (Fig. 1; Okuda and Takahashi, 1962a; Horiguchi, 1988; Li *et al.*, 2012), but the exact processes underlying this Si effect are still unknown. Several possible mechanisms have been proposed including the precipitation of Mn on the root surface due to Si-increased oxidation (Okuda and Takahashi, 1962a), enhanced internal tolerance to Mn (Horiguchi, 1988) and inhibited root-to-shoot translocation of Mn (Li *et al.*, 2012). However, the evidence supporting these possible mechanisms is not convincing enough. In the present study, we took the advantage of using a rice mutant (*lsi1*) defective in Si uptake to study the interaction between Si and Mn in rice. This mutant has a mutation in Si influx transporter Lsi1, which is localized at the distal side of both the exodermis and endodermis of the roots (Ma *et al.*, 2006). Lsi1 is responsible for the transport of Si from the external solution to the root cells, therefore, knockout of this transporter gene resulted in loss of Si uptake (Ma *et al.*, 2006). We found that, in contrast to its effect on the WT, Si supply did not alleviate Mn toxicity in the *lsi1* mutant (Fig. 1C). The Mn concentration in the shoot was decreased by Si in the WT, but not in the mutant (Fig. 2A, B), which is consistent with the observed Mn toxicity symptoms. These results suggest that Si uptake by the roots is important for the alleviation of Mn toxicity. By comparing this mutant with its wild-type rice, we found that the Si-decreased Mn toxicity results from both reduced uptake and root-to-shoot translocation of Mn.

### Si reduced Mn uptake by down-regulating the Mn transporter, OsNramp5

Silicon decreased Mn uptake in the WT, but not in the *lsi1* mutant (Fig. 3A, B). This result in the WT is consistent with previous studies (e.g. Okuda and Takahashi, 1962a; Horiguchi, 1988; Li *et al.*, 2012). Okuda and Takahashi (1962a) speculated that this Si-decreased Mn uptake was due to Si-enhanced oxidizing capacity of rice roots, resulting in Mn^2+^ oxidation on the root surface, thereby suppressing its uptake. However, our results did not support this speculation. Rice is able to accumulate Mn at a high concentration in the shoots and >90% of total Mn taken up was translocated to the shoots (Fig. 3). The shoots accumulated much higher Mn than the roots in both the WT and mutant in the absence and presence of Si (Fig. 3A, B). Therefore, retention of Mn in the roots due to oxidation did not account for the large decrease of Mn accumulation in the shoots. This is different from Fe, whose toxicity could be also alleviated by Si (Okuda and Takahashi, 1962b). Most Fe is retained in the roots due to Si-enhanced oxidation of ferrous Fe to ferric Fe, suppressing its uptake to the root cells. In fact, a short-term experiment clearly shows that the total Mn uptake was unaffected by the coexistence of Si (Fig. 5A).

Our results indicate that Si-decreased Mn uptake over the relative long-term is attributed to the down-regulation of *OsNramp5* expression (Fig. 8). OsNramp5 is a major transporter for Mn uptake in rice (Sasaki *et al.*, 2012). Similar to Lsi1 (Ma *et al.*, 2006), OsNramp5 is polarly localized at the distal side of both exodermis and endodermis of the roots (Sasaki *et al.*, 2012), which is responsible for transport of Mn from the external solution to the root cells. The expression of *OsNramp5* was not affected by short exposure to Si (1 d), but was down-regulated by longer exposure (3 d) (Fig. 8). This regulation pattern of *OsNramp5* is quite similar to that of *Lsi1* and *Lsi2* to Si (Ma *et al.*, 2006, 2007). Furthermore, this result is also consistent with that of Mn uptake; Mn uptake was not affected by short-term exposure to Si (Fig. 5), but decreased with relatively long-term exposure to Si (Fig. 3). This is also supported by the fact that pretreatment with Si decreased Mn uptake (Fig. 6). Interestingly, both the expression of *OsNramp5* and Mn uptake did not respond to Si in the *lsi1* mutant (Fig. 8). These results indicate that Si-decreased Mn uptake during the longer-term treatment with Si is due to suppressed expression of *OsNramp5*. The expression of *OsNramp5* did not respond to Mn concentrations (Sasaki *et al.*, 2012). In this study, the expression level at both 0.5 and 200 µM Mn supply was also similar (Fig. 8). This suggests that the down-regulation of *OsNramp5* is not caused by Si-induced Mn change, but by Si accumulation in the tissues; although the exact mechanisms for this remains to be examined in future.

### Silicon reduced root-to-shoot translocation of Mn probably due to formation of Si-Mn complex

Silicon did not alter Mn accumulation in the cell wall (Fig. 7C), but decreased the root-to-shoot translocation rate of Mn in the WT (Fig. 3C). This is consistent with Si-increased Mn in the root cell sap and Si-decreased Mn in the xylem sap in the WT (Fig. 7A, D). A short-term uptake experiment also revealed that the translocation of ∆Mn was rapidly reduced by the coexistence of Si in the WT (Fig. 5). Since similar phenomena were not observed in the *lsi1* mutant, an interaction of Si-Mn within the root cells may be responsible for this Si effect. Mn taken up by OsNramp5 at the distal side of the exodermis and endodermis must be released from the cells toward the stele for subsequent xylem loading. This process requires an efflux transporter as seen in the Si transport system (Ma *et al.*, 2007; Ma and Yamaji, 2015). Recently, an efflux transporter (OsMTP9) for Mn has been identified (Ueno *et al.*, 2015); there is a possibility that this transporter is also down-regulated by Si directly or indirectly. This is supported by the fact that in the *lsi1* mutant with a very low internal Si concentration, the root-to-shoot translocation of Mn was not affected by Si (Fig. 3C).

Another explanation for the Mn-Si interaction is the formation of a Mn-Si complex. Although information on a Mn-Si complex is not available, formation of a Zn-silicate complex has been suggested to be responsible for the amelioration of the Zn toxicity in *Cardaminopsis* (Neumann and Zurnieden, 2001). In addition, formation of Al-Si and Cd-Si complexes has also been reported to be involved in the detoxification of Al and Cd (Wang *et al.*, 2004; Liu *et al.*, 2013; Ma *et al.*, 2015). Since OsMTP9 transports ionic Mn (Ueno *et al.*, 2015), the Si-Mn complex will not be transported to the stele for subsequent root-to-shoot translocation, resulting in Si remaining in the root cells. This is supported by the observation that the Si:Mn stoichiometric ratio in the root cell sap was similar in both the WT and *lsi1* mutant. The Si concentration in the root cell sap of the mutant was higher than the accumulation of Si in the shoots (Fig. 7B; Ma *et al.*, 2002). This may be attributed to another Si transporter, Lsi6, which is expressed in the root tips (Yamaji *et al.*, 2008). But this transporter did not contribute to the root-to-shoot translocation of Si. Therefore, the Si concentration of the cell sap in the mature root region would be much lower in the *lsi1* mutant. In addition, it seems that the formation of a Mn-Si complex is very rapid because Mn concentration in the root cell sap was increased after only 3h exposure to Si (Fig. 7E), a property that requires future examination.

In conclusion, our results indicate that the Si-decreased Mn accumulation in rice is a consequence of both reduced root-to-shoot translocation of Mn, probably by formation of Mn-Si complex in the root cytosol, and decreased Mn uptake due to down-regulation of *OsNramp5* gene in rice.
